# (2*S*,4*R*)-2-[(1*R*)-1-(4-Bromo­phen­yl)-2-nitro­eth­yl]-4-ethyl­cyclo­hexa­none

**DOI:** 10.1107/S1600536813001426

**Published:** 2013-01-19

**Authors:** Chi-Xiao Zhang, Yan-Peng Zhang, Ai-Bao Xia

**Affiliations:** a State Key Laboratory Breeding Base of Green Chemistry-Synthesis Technology, Zhejiang University of Technology, Hangzhou 310014, People’s Republic of China

## Abstract

The crystal structure of the title compound, C_16_H_20_BrNO_3_, contains three chiral centers in the configuration 1*R*,2*S*,6*R*. The cyclo­hexane ring is in a chair conformation. In the crystal, mol­ecules are linked by weak C—H⋯O inter­actions, forming chains along the *a*-axis direction.

## Related literature

For related compounds, see: Hayashi *et al.* (2005 ▶); Li *et al.* (2009 ▶); Xia *et al.* (2009 ▶); Wu *et al.* (2011 ▶). For asymmetric Michael addition reactions, see: Luo *et al.* (2007 ▶). For enanti­oselective organocatalytic Michael additions, see: Peelen *et al.* (2005 ▶); Ma *et al.* (2008 ▶).
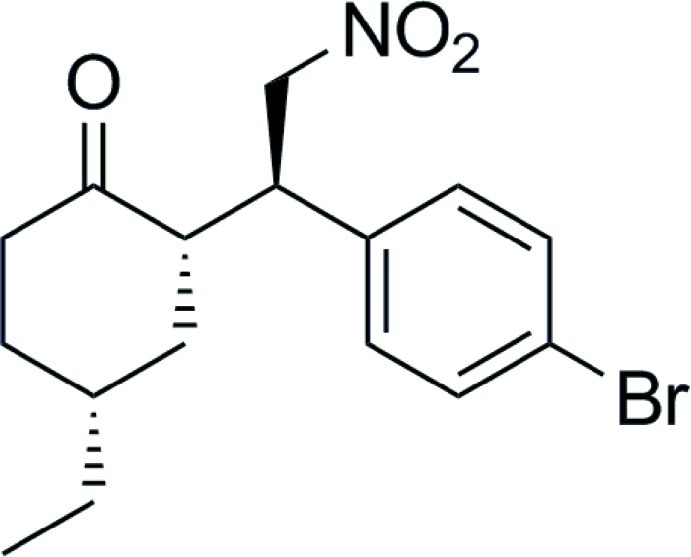


## Experimental

### 

#### Crystal data

C_16_H_20_BrNO_3_
*M* *_r_* = 354.24
Monoclinic, 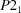

*a* = 5.6434 (4) Å
*b* = 9.2179 (6) Å
*c* = 16.5472 (9) Å
β = 101.782 (3)°
*V* = 842.65 (9) Å^3^
*Z* = 2
Mo *K*α radiation
μ = 2.45 mm^−1^
*T* = 296 K
0.52 × 0.31 × 0.18 mm

#### Data collection

Rigaku R-AXIS RAPID/ZJUG diffractometer
Absorption correction: multi-scan (*ABSCOR*; Higashi, 1995 ▶) *T*_min_ = 0.279, *T*_max_ = 0.644
7234 measured reflections
3277 independent reflections
1619 reflections with *I* > 2σ(*I*)
*R*_int_ = 0.071

#### Refinement

*R*[*F* ^2^ > 2σ(*F* ^2^)] = 0.056
*wR*(*F* ^2^) = 0.175
*S* = 1.00
3277 reflections
191 parameters
1 restraint
H-atom parameters constrained
Δρ_max_ = 0.35 e Å^−3^
Δρ_min_ = −0.29 e Å^−3^
Absolute structure: Flack (1983 ▶), 1259 Friedel pairs
Flack parameter: 0.03 (2)

### 

Data collection: *PROCESS-AUTO* (Rigaku, 2006 ▶); cell refinement: *PROCESS-AUTO*; data reduction: *CrystalStructure* (Rigaku, 2007 ▶); program(s) used to solve structure: *SHELXS97* (Sheldrick, 2008 ▶); program(s) used to refine structure: *SHELXL97* (Sheldrick, 2008 ▶); molecular graphics: *ORTEP-3 for Windows* (Farrugia, 2012 ▶); software used to prepare material for publication: *WinGX* (Farrugia, 2012 ▶).

## Supplementary Material

Crystal structure: contains datablock(s) global, I. DOI: 10.1107/S1600536813001426/zq2194sup1.cif

Structure factors: contains datablock(s) I. DOI: 10.1107/S1600536813001426/zq2194Isup2.hkl

Supplementary material file. DOI: 10.1107/S1600536813001426/zq2194Isup3.cml

Additional supplementary materials:  crystallographic information; 3D view; checkCIF report

## Figures and Tables

**Table 1:** Hydrogen-bond geometry (Å, °)

*D*—H⋯*A*	*D*—H	H⋯*A*	*D*⋯*A*	*D*—H⋯*A*
C11—H11⋯O3^i^	0.93	2.56	3.478 (9)	169
C16—H16*B*⋯O3^i^	0.97	2.58	3.500 (8)	158

Symmetry code: (i) 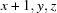.

**Fig. 1.**
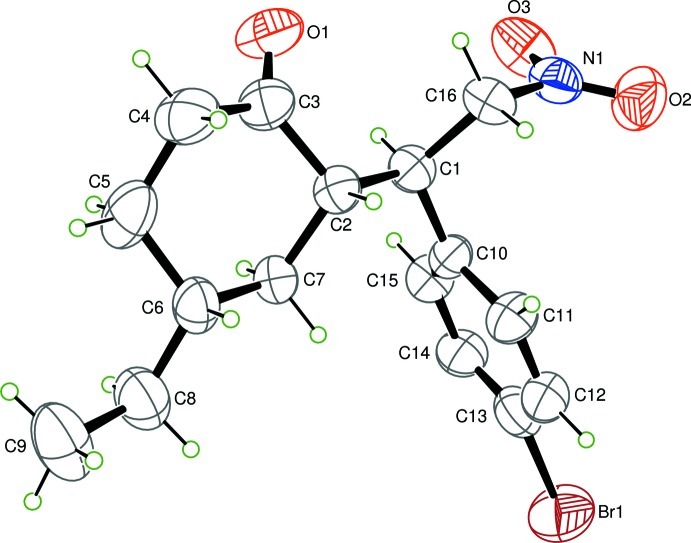
The molecular structure of the title compound with the atomic labeling scheme. Displacement ellipsoids are drawn at the 50% probability level.

## supplementary crystallographic information

### Comment

The Michael reaction (e.g. Luo *et al.*, 2007; Ma *et al.*, 2008; Peelen *et al.*, 2005) of a carbon nucleophile with a nitroalkene is one useful synthetic method for the preparation of nitroalkanes, which are versatile synthetic intermediates owing to the various possible transformations of the nitro group into other useful functional groups (Hayashi *et al.*, 2005). The title compound was obtained from the Michael addition of 4-ethylcyclohexanone to 1-(4-bromophenyl)-2-nitroethene in our laboratory. For related structures, see: Li *et al.* (2009); Xia *et al.* (2009); Wu *et al.* (2011).

The title compound is shown in Fig. 1.The cyclohexyl ring adopts a chair conformation. The plane of the phenyl ring and the least-square plane of the cyclohexyl moiety enclose an angle of 70.00 (3)° while the plane through the nitro group and the adjacent C16 atom encloses an angle of 63.26 (3)° with the phenyl ring. In the crystal, molecules are linked by weak C—H···O interactions forming chains along the *a* axis (Table 1).

### Experimental

An isopropyl ether (0.5 mL) solution of 1-(4-bromophenyl)-2-nitroethene (0.6 mmol) and 4-ethylcyclohexanone (1.2 mmol) was stirred with the (*S*)-2-(pyrrolidin-2-ylmethylthio)pyridine (0.12 mmol) as catalyst and benzoic acid (0.12 mmol) as cocatalyst at room temperature. After completion of the reaction, the mixture was extracted with ethyl acetate. Solvents were removed under vacuum and the residue was purified by column chromatography on silica gel (eluent: petroleum ether-ether). Suitable crystals were obtained by slow evaporation of an ether solution.

### Refinement

H atoms were placed in calculated position with C—H ranging from 0.93 Å to 0.98 Å. All H atoms were included in the final cycles of refinement as riding mode, with *U*_iso_(H) = 1.2*U*_eq_(C) for aromatic, methylene and methine H atoms, and with *U*_iso_(H) = 1.5*U*_eq_(C) for methyl H atoms.

### Crystal data

**Table:**

C_16_H_20_BrNO_3_	*F*(000) = 364
*M**_r_* = 354.24	*D*_x_ = 1.396 Mg m^−^^3^
Monoclinic, *P*2_1_	Mo *K*α radiation, λ = 0.71073 Å
Hall symbol: P 2yb	Cell parameters from 4682 reflections
*a* = 5.6434 (4) Å	θ = 3.4–27.4°
*b* = 9.2179 (6) Å	µ = 2.45 mm^−^^1^
*c* = 16.5472 (9) Å	*T* = 296 K
β = 101.782 (3)°	Platelet, colourless
*V* = 842.65 (9) Å^3^	0.52 × 0.31 × 0.18 mm
*Z* = 2	

### Data collection

**Table:**

Rigaku R-AXIS RAPID/ZJUG diffractometer	3277 independent reflections
Radiation source: rotating anode	1619 reflections with *I* > 2σ(*I*)
Graphite monochromator	*R*_int_ = 0.071
Detector resolution: 10.00 pixels mm^-1^	θ_max_ = 26.0°, θ_min_ = 3.4°
ω scans	*h* = −6→6
Absorption correction: multi-scan (*ABSCOR*; Higashi, 1995)	*k* = −11→11
*T*_min_ = 0.279, *T*_max_ = 0.644	*l* = −20→20
7234 measured reflections	

### Refinement

**Table:**

Refinement on *F*^2^	Hydrogen site location: inferred from neighbouring sites
Least-squares matrix: full	H-atom parameters constrained
*R*[*F*^2^ > 2σ(*F*^2^)] = 0.056	*w* = 1/[σ^2^(*F*_o_^2^) + (0.0605*P*)^2^ + 0.3*P*] where *P* = (*F*_o_^2^ + 2*F*_c_^2^)/3
*wR*(*F*^2^) = 0.175	(Δ/σ)_max_ < 0.001
*S* = 1.00	Δρ_max_ = 0.35 e Å^−^^3^
3277 reflections	Δρ_min_ = −0.29 e Å^−^^3^
191 parameters	Extinction correction: *SHELXL97* (Sheldrick, 2008), Fc^*^=kFc[1+0.001xFc^2^λ^3^/sin(2θ)]^-1/4^
1 restraint	Extinction coefficient: 0.072 (8)
Primary atom site location: structure-invariant direct methods	Absolute structure: Flack (1983), 1259 Friedel pairs
Secondary atom site location: difference Fourier map	Flack parameter: 0.03 (2)

### Special details

**Table:**

Geometry. All e.s.d.'s (except the e.s.d. in the dihedral angle between two l.s. planes) are estimated using the full covariance matrix. The cell e.s.d.'s are taken into account individually in the estimation of e.s.d.'s in distances, angles and torsion angles; correlations between e.s.d.'s in cell parameters are only used when they are defined by crystal symmetry. An approximate (isotropic) treatment of cell e.s.d.'s is used for estimating e.s.d.'s involving l.s. planes.
Refinement. Refinement of *F*^2^ against ALL reflections. The weighted *R*-factor *wR* and goodness of fit *S* are based on *F*^2^, conventional *R*-factors *R* are based on *F*, with *F* set to zero for negative *F*^2^. The threshold expression of *F*^2^ > σ(*F*^2^) is used only for calculating *R*-factors(gt) *etc*. and is not relevant to the choice of reflections for refinement. *R*-factors based on *F*^2^ are statistically about twice as large as those based on *F*, and *R*- factors based on ALL data will be even larger.

### Fractional atomic coordinates and isotropic or equivalent isotropic displacement parameters (Å^2^)

**Table:**

	*x*	*y*	*z*	*U*_iso_*/*U*_eq_	
C1	0.2312 (11)	0.2884 (7)	0.1770 (4)	0.0563 (15)	
H1	0.0766	0.3379	0.1754	0.068*	
C2	0.4174 (11)	0.4066 (6)	0.1660 (4)	0.0541 (15)	
H2	0.5499	0.3592	0.1461	0.065*	
C3	0.3093 (14)	0.5225 (7)	0.1030 (4)	0.0712 (19)	
C4	0.4813 (15)	0.6373 (10)	0.0880 (5)	0.091 (3)	
H4A	0.3957	0.7096	0.0504	0.109*	
H4B	0.6060	0.5948	0.0629	0.109*	
C5	0.5974 (14)	0.7093 (9)	0.1702 (5)	0.090 (2)	
H5A	0.7190	0.7778	0.1605	0.108*	
H5B	0.4742	0.7634	0.1905	0.108*	
C6	0.7146 (11)	0.6025 (7)	0.2361 (5)	0.0672 (18)	
H6	0.8446	0.5533	0.2156	0.081*	
C7	0.5264 (10)	0.4859 (8)	0.2467 (3)	0.0582 (14)	
H7A	0.6031	0.4153	0.2872	0.070*	
H7B	0.3969	0.5317	0.2679	0.070*	
C8	0.8263 (14)	0.6704 (9)	0.3179 (5)	0.089 (2)	
H8A	0.8867	0.5934	0.3565	0.107*	
H8B	0.6999	0.7206	0.3387	0.107*	
C9	1.0317 (16)	0.7769 (10)	0.3167 (7)	0.110 (3)	
H9A	1.0904	0.8137	0.3714	0.165*	
H9B	0.9736	0.8558	0.2802	0.165*	
H9C	1.1607	0.7282	0.2978	0.165*	
C10	0.2948 (10)	0.2114 (7)	0.2592 (4)	0.0522 (14)	
C11	0.4955 (12)	0.1193 (7)	0.2768 (4)	0.0646 (17)	
H11	0.5875	0.1042	0.2369	0.078*	
C12	0.5606 (13)	0.0500 (7)	0.3524 (5)	0.075 (2)	
H12	0.6965	−0.0094	0.3637	0.090*	
C13	0.4189 (15)	0.0708 (8)	0.4112 (5)	0.076 (2)	
C14	0.2187 (14)	0.1583 (8)	0.3947 (4)	0.0708 (19)	
H14	0.1244	0.1714	0.4341	0.085*	
C15	0.1579 (11)	0.2267 (8)	0.3194 (4)	0.0622 (17)	
H15	0.0206	0.2851	0.3085	0.075*	
C16	0.1937 (11)	0.1852 (7)	0.1032 (4)	0.0616 (17)	
H16A	0.1394	0.2399	0.0527	0.074*	
H16B	0.3464	0.1395	0.1001	0.074*	
N1	0.0135 (12)	0.0724 (7)	0.1101 (4)	0.0700 (16)	
O1	0.0953 (10)	0.5240 (6)	0.0704 (3)	0.0925 (19)	
O2	0.0723 (11)	−0.0541 (7)	0.1111 (4)	0.1028 (18)	
O3	−0.1912 (9)	0.1117 (7)	0.1163 (3)	0.0939 (17)	
Br1	0.5073 (2)	−0.02467 (17)	0.51442 (5)	0.1269 (6)	

### Atomic displacement parameters (Å^2^)

**Table:**

	*U*^11^	*U*^22^	*U*^33^	*U*^12^	*U*^13^	*U*^23^
C1	0.058 (3)	0.059 (3)	0.054 (4)	−0.002 (3)	0.015 (3)	−0.004 (3)
C2	0.053 (3)	0.055 (3)	0.056 (3)	−0.005 (3)	0.015 (3)	0.006 (3)
C3	0.089 (5)	0.074 (5)	0.053 (4)	−0.003 (4)	0.019 (3)	0.010 (3)
C4	0.096 (5)	0.099 (6)	0.074 (5)	−0.021 (5)	0.007 (4)	0.028 (4)
C5	0.084 (5)	0.071 (5)	0.120 (7)	−0.004 (4)	0.028 (5)	0.035 (5)
C6	0.058 (4)	0.052 (4)	0.091 (5)	−0.004 (3)	0.012 (4)	−0.001 (4)
C7	0.063 (3)	0.050 (3)	0.059 (3)	0.002 (3)	0.008 (3)	−0.005 (4)
C8	0.084 (5)	0.076 (5)	0.105 (6)	−0.013 (4)	0.013 (4)	−0.015 (4)
C9	0.087 (5)	0.092 (6)	0.153 (10)	−0.022 (5)	0.030 (6)	−0.035 (6)
C10	0.050 (3)	0.050 (3)	0.056 (4)	0.002 (3)	0.009 (3)	0.005 (3)
C11	0.072 (4)	0.067 (4)	0.059 (4)	0.004 (4)	0.023 (3)	0.010 (3)
C12	0.069 (4)	0.061 (4)	0.089 (5)	0.005 (3)	0.000 (4)	−0.002 (4)
C13	0.094 (5)	0.069 (4)	0.063 (4)	−0.006 (4)	0.011 (4)	−0.015 (4)
C14	0.085 (5)	0.083 (5)	0.047 (4)	0.001 (4)	0.020 (3)	−0.003 (3)
C15	0.062 (4)	0.066 (4)	0.062 (4)	0.001 (4)	0.019 (3)	0.002 (3)
C16	0.060 (3)	0.074 (5)	0.051 (3)	−0.011 (3)	0.012 (3)	−0.008 (4)
N1	0.077 (4)	0.078 (5)	0.053 (3)	−0.015 (4)	0.009 (3)	−0.001 (3)
O1	0.081 (3)	0.111 (5)	0.077 (3)	0.000 (3)	−0.003 (3)	0.031 (3)
O2	0.107 (4)	0.067 (4)	0.128 (5)	−0.012 (3)	0.007 (3)	0.016 (3)
O3	0.063 (3)	0.127 (5)	0.097 (4)	−0.028 (3)	0.030 (3)	−0.031 (3)
Br1	0.1788 (11)	0.1258 (9)	0.0677 (6)	0.0169 (8)	0.0050 (5)	0.0258 (7)

### Geometric parameters (Å, º)

**Table:**

C1—C10	1.511 (8)	C8—H8A	0.9700
C1—C16	1.528 (8)	C8—H8B	0.9700
C1—C2	1.550 (8)	C9—H9A	0.9600
C1—H1	0.9800	C9—H9B	0.9600
C2—C3	1.530 (8)	C9—H9C	0.9600
C2—C7	1.535 (8)	C10—C15	1.387 (8)
C2—H2	0.9800	C10—C11	1.398 (8)
C3—O1	1.218 (8)	C11—C12	1.386 (9)
C3—C4	1.490 (10)	C11—H11	0.9300
C4—C5	1.536 (12)	C12—C13	1.392 (11)
C4—H4A	0.9700	C12—H12	0.9300
C4—H4B	0.9700	C13—C14	1.370 (10)
C5—C6	1.517 (10)	C13—Br1	1.896 (8)
C5—H5A	0.9700	C14—C15	1.376 (9)
C5—H5B	0.9700	C14—H14	0.9300
C6—C8	1.506 (10)	C15—H15	0.9300
C6—C7	1.546 (9)	C16—N1	1.475 (9)
C6—H6	0.9800	C16—H16A	0.9700
C7—H7A	0.9700	C16—H16B	0.9700
C7—H7B	0.9700	N1—O2	1.212 (9)
C8—C9	1.522 (11)	N1—O3	1.235 (7)
			
C10—C1—C16	113.3 (5)	C6—C8—C9	115.9 (8)
C10—C1—C2	113.2 (5)	C6—C8—H8A	108.3
C16—C1—C2	109.3 (5)	C9—C8—H8A	108.3
C10—C1—H1	106.9	C6—C8—H8B	108.3
C16—C1—H1	106.9	C9—C8—H8B	108.3
C2—C1—H1	106.9	H8A—C8—H8B	107.4
C3—C2—C7	107.1 (5)	C8—C9—H9A	109.5
C3—C2—C1	112.7 (5)	C8—C9—H9B	109.5
C7—C2—C1	113.2 (5)	H9A—C9—H9B	109.5
C3—C2—H2	107.9	C8—C9—H9C	109.5
C7—C2—H2	107.9	H9A—C9—H9C	109.5
C1—C2—H2	107.9	H9B—C9—H9C	109.5
O1—C3—C4	122.6 (6)	C15—C10—C11	117.3 (6)
O1—C3—C2	122.0 (6)	C15—C10—C1	122.3 (5)
C4—C3—C2	115.3 (6)	C11—C10—C1	120.4 (5)
C3—C4—C5	109.5 (6)	C12—C11—C10	121.5 (6)
C3—C4—H4A	109.8	C12—C11—H11	119.2
C5—C4—H4A	109.8	C10—C11—H11	119.2
C3—C4—H4B	109.8	C11—C12—C13	118.9 (7)
C5—C4—H4B	109.8	C11—C12—H12	120.6
H4A—C4—H4B	108.2	C13—C12—H12	120.6
C6—C5—C4	113.7 (7)	C14—C13—C12	120.7 (7)
C6—C5—H5A	108.8	C14—C13—Br1	120.5 (6)
C4—C5—H5A	108.8	C12—C13—Br1	118.8 (6)
C6—C5—H5B	108.8	C15—C14—C13	119.6 (7)
C4—C5—H5B	108.8	C15—C14—H14	120.2
H5A—C5—H5B	107.7	C13—C14—H14	120.2
C8—C6—C5	114.6 (6)	C14—C15—C10	122.1 (6)
C8—C6—C7	111.0 (6)	C14—C15—H15	119.0
C5—C6—C7	108.9 (5)	C10—C15—H15	119.0
C8—C6—H6	107.3	N1—C16—C1	111.2 (5)
C5—C6—H6	107.3	N1—C16—H16A	109.4
C7—C6—H6	107.3	C1—C16—H16A	109.4
C2—C7—C6	113.3 (5)	N1—C16—H16B	109.4
C2—C7—H7A	108.9	C1—C16—H16B	109.4
C6—C7—H7A	108.9	H16A—C16—H16B	108.0
C2—C7—H7B	108.9	O2—N1—O3	122.6 (7)
C6—C7—H7B	108.9	O2—N1—C16	119.2 (7)
H7A—C7—H7B	107.7	O3—N1—C16	118.1 (6)
			
C10—C1—C2—C3	−156.6 (5)	C16—C1—C10—C15	−122.7 (7)
C16—C1—C2—C3	76.1 (6)	C2—C1—C10—C15	112.1 (7)
C10—C1—C2—C7	−34.9 (7)	C16—C1—C10—C11	56.5 (8)
C16—C1—C2—C7	−162.2 (5)	C2—C1—C10—C11	−68.7 (8)
C7—C2—C3—O1	−120.3 (7)	C15—C10—C11—C12	−2.2 (9)
C1—C2—C3—O1	4.8 (9)	C1—C10—C11—C12	178.6 (6)
C7—C2—C3—C4	56.5 (8)	C10—C11—C12—C13	1.3 (10)
C1—C2—C3—C4	−178.4 (6)	C11—C12—C13—C14	0.0 (11)
O1—C3—C4—C5	121.3 (8)	C11—C12—C13—Br1	179.8 (5)
C2—C3—C4—C5	−55.4 (9)	C12—C13—C14—C15	−0.3 (11)
C3—C4—C5—C6	53.7 (9)	Br1—C13—C14—C15	179.9 (6)
C4—C5—C6—C8	−179.0 (6)	C13—C14—C15—C10	−0.7 (11)
C4—C5—C6—C7	−54.0 (8)	C11—C10—C15—C14	1.9 (10)
C3—C2—C7—C6	−56.0 (7)	C1—C10—C15—C14	−178.9 (6)
C1—C2—C7—C6	179.2 (5)	C10—C1—C16—N1	54.0 (7)
C8—C6—C7—C2	−176.6 (5)	C2—C1—C16—N1	−178.8 (5)
C5—C6—C7—C2	56.3 (8)	C1—C16—N1—O2	−120.9 (8)
C5—C6—C8—C9	−61.7 (9)	C1—C16—N1—O3	57.6 (7)
C7—C6—C8—C9	174.4 (7)		

### Hydrogen-bond geometry (Å, º)

**Table:**

*D*—H···*A*	*D*—H	H···*A*	*D*···*A*	*D*—H···*A*
C11—H11···O3^i^	0.93	2.56	3.478 (9)	169
C16—H16*B*···O3^i^	0.97	2.58	3.500 (8)	158

Symmetry code: (i) *x*+1, *y*, *z*.
